# Transition of patients with hereditary nephropathies from paediatric to adult care

**DOI:** 10.1093/ndt/gfaf186

**Published:** 2025-09-10

**Authors:** Maria Vanessa Perez Gomez, George-Claudiu Costea, Laura Claus, Emilie Cornec-Le Gall, Albertien M van Eerde, Sandrine Lemoine, Jaap Groothoff, Elena Levtchenko, Luisa Klein, Lars Pape, Roman-Ulrich Müller, Max C Liebau

**Affiliations:** Department of Nephrology and Hypertension, Health Research Institute-Fundación Jiménez Díaz University Hospital, Universidad Autónoma de Madrid (IIS-FJD, UAM), Madrid, Spain; Department of Nephrology and Hypertension, Hospital Universitario Fundación Jiménez Díaz, Madrid, Spain; RICORS2040, Madrid, Spain; Pediatric Nephrology Department, Fundeni Clinical Institute, Bucharest, Romania; Department of Genetics, University Medical Centre Utrecht, Utrecht, The Netherlands; Service de Néphrologie, Hémodialyse et Transplantation Rénale, Centre de référence MARHEA, Filière ORKID, CHRU Brest, Brest, France; University of Brest, Inserm, UMR 1078, GGB, Brest, France; Department of Genetics, University Medical Centre Utrecht, Utrecht, The Netherlands; Service de Néphrologie, Dialyse, Exploration Fonctionnelle Rénale, Hôpital Edouard Herriot, HCL, INSERM 1060, Lyon, France; Department of Paediatric Nephrology, Emma Children’s Hospital, Amsterdam UMC, Amsterdam, The Netherlands; Department of Paediatric Nephrology, Emma Children’s Hospital, Amsterdam UMC, Amsterdam, The Netherlands; Division of Pediatric Nephrology, Department of Pediatrics, University Hospital Cologne and Faculty of Medicine, University of Cologne, Cologne, Germany; Department of Pediatrics, University Medical Hospital, Essen, Germany; Department II of Internal Medicine, Faculty of Medicine and University Hospital, University of Cologne, Cologne, Germany; Center for Rare Diseases Cologne, Faculty of Medicine and University Hospital Cologne, University of Cologne, Cologne, Germany; Cologne Excellence Cluster on Cellular Stress Responses in Aging-Associated Diseases (CECAD), Cologne, Germany; Department of Pediatrics, RWTH Aachen University Hospital, Aachen, Germany; Department of Pediatrics and Center for Family Health, University Hospital Cologne and Faculty of Medicine, University of Cologne, Cologne, Germany; Center for Rare Diseases, University Hospital Cologne and Faculty of Medicine, University of Cologne, Cologne, Germany; Center for Molecular Medicine, University Hospital Cologne and Faculty of Medicine, University of Cologne, Cologne, Germany

**Keywords:** chronic kidney disease, genetic kidney disease, inherited kidney disease, transfer, transition

## Abstract

Adolescents and young adults with chronic kidney disease (CKD), particularly those with genetic kidney diseases, face unique challenges as they transition from paediatric to adult nephrology care. This period is marked not only by changes in healthcare providers but also by significant developmental, psychosocial and medical complexities. In response, the European Renal Association Working Group on Genes and Kidney and the European Society for Paediatric Nephrology Working Group on Inherited Kidney Diseases have collaborated to develop practical advice for healthcare professionals involved in transition care across Europe and beyond. This document outlines key principles and offers practical recommendations to support a successful transition, emphasizing the need for early planning, patient education, individualized approaches and multidisciplinary coordination. Special considerations are highlighted for patients with genetic kidney diseases, including those with syndromic manifestations, reproductive implications and the need for continuity of care across specialties. The document also identifies knowledge gaps, proposes directions for future research and collaboration and encourages the implementation of transition protocols adapted to national and local healthcare systems. By harmonizing practices and fostering shared responsibility between paediatric and adult nephrology teams, this joint initiative aims to improve health outcomes, patient empowerment and long-term engagement in care for young people with CKD.

KEY LEARNING POINTS **What was known:**

- Transition from paediatric to adult nephrology is challenging, especially in patients with hereditary kidney diseases.
- Most existing transition protocols have been developed within paediatric services, with limited involvement of adult nephrologists.
- There was no joint European guidance integrating genetic aspects into the transition process for hereditary nephropathies.

**This study adds:**

- This article provides expert-based, practical advice jointly developed by the European Renal Association and the European Society for Paediatric Nephrology on the transition of patients with hereditary kidney diseases.
- It outlines key elements, including genetic counselling, structured planning and disease-specific considerations.
- It bridges the gap between paediatric and adult nephrology, incorporating multidisciplinary and genetic perspectives into transition care.

**Potential impact:**

- This guidance will support the development of structured transition programs tailored to inherited kidney diseases.
- It promotes adult nephrologist involvement and awareness of the genetic complexity of these conditions.
- It may contribute to more consistent care pathways, improved adherence and better outcomes for transitioning patients.

## INTRODUCTION

The transition from paediatric to adult care for patients with chronic kidney disease (CKD) poses unique challenges. Patients with hereditary and congenital forms of CKD, whether confirmed or suspected, make up the largest proportion of patients undergoing transition. These patients face specific difficulties due to the chronic nature of their disease, the possible presence of extrarenal manifestations and, for a subgroup, concerns about the risk of passing the disease to their future children. Their disease often requires lifelong management and can affect various aspects of their lives, including psychological well-being, social interactions and educational progress and may warrant genetic counselling before parenthood. This article aims to offer practical advice to adult and paediatric nephrologists on how to manage the transition of patients with hereditary nephropathies, drawing from current evidence and expert consensus. The practical advice provided seeks to support continuity of care, reduce the risk of loss to follow-up, improve adherence and promote appropriate integration of genetic (re)evaluation, ultimately contributing to better health outcomes.

## TRANSITION PROCESS OVERVIEW

In this consensus statement, we define ‘transition’ as the process of preparing a paediatric patient for the eventual ‘transfer’ to adult care followed by further support during the first period after this transfer. Transition includes education, psychological preparation and the gradual handover of responsibility from caregivers and parents to the patient, with the goal of promoting the highest possible level of autonomy in managing their condition within the adult healthcare setting. However, we acknowledge that some patients, particularly those with developmental or cognitive impairments, will require ongoing involvement of parents or caregivers after transfer. In such cases, appropriate legal arrangements may be needed to support shared decision-making in the adult care setting, where parents should remain actively engaged as part of the care team.

In contrast, ‘transfer’ refers to the specific moment when paediatric care ends and adult care formally begins. It is important to distinguish between these two concepts because transition represents a longer, more comprehensive process that supports the transfer event, ensures continuity of care and minimizes the risks associated with the transition.

The transition for patients with hereditary kidney diseases often involves unique complexities that go beyond the usual scope of care. It involves the patient, the family and/or caregivers and healthcare professionals from both paediatric and adult specialties. In addition to managing their kidney disease, the patients must also be prepared to deal with the broader implications of their genetic diagnosis when applicable, including the potential impact on future family planning. A multidisciplinary approach is often necessary to ensure the success of this process. Evidence-based general guidelines for transition have been published before that could serve as a fundament for disease-specific guidelines [1].

The optimal age for initiating transition remains a topic of debate. Many professional societies recommend starting a transition plan early, ideally by the age of 12–14 years, or at least 1 year before the actual transfer [2, 3]. The transfer is usually finalized in most situations by the age of 18, although in some healthcare systems and for specific patients it can occur later, with a reported range of 16–25 years in different settings [2]. The process ideally occurs gradually when the patient is ready, e.g. after completing growth and puberty, and once the disease is stable [4]. The concept of transition can be introduced to the patient and family members or caregivers by any member of the multidisciplinary paediatric team, including physicians, nurses or other health professionals involved in the patient’s care. However, the paediatric doctor is usually responsible for coordinating the process and ensuring it is addressed. Although the transition process should take into account the patient’s individual level of understanding and acceptance, the decision to transfer is not always voluntary and is often determined by institutional or systemic constraints. Following careful transition can help reduce the risk of patients becoming lost to follow-up.

It is important to involve the patient’s family and/or caregivers in the transition process. They can be provided with tools to help them overcome their fear of losing control over the disease, while gradually encouraging the patient to take more autonomy and responsibility [3].

As part of the transition process, we recommend considering a genetic evaluation in all patients with confirmed or suspected hereditary CKD. This includes reviewing the patient’s phenotypic traits and family history and referring for genetic counselling and/or testing when appropriate. These aspects are further developed in detail in Genetic Diagnosis and Role of the Geneticist in Transition. For a concise summary of practical advice related to the transition process overview, please refer to Box 1.

Box 1: **Transition process overview—practical advice.**

1.1. Early planning: Initiate the transition plan early, preferentially by the age of 14, or at least 1 year before the actual transfer.

1.2. Gradual transition: Transition is ideally gradual, with the transfer to adult nephrology occurring when the patient is ready, has finished growing, has completed puberty and when the disease is stable.

1.3. Patient-centred approach: The transition is best approached as a voluntary and well-understood decision by the patient, involving the family and caregivers. Tools can be provided to help the family gradually transfer more autonomy and responsibility to the patient.

1.4. Genetic consideration: Consider genetic evaluation during transition in patients with confirmed or suspected hereditary CKD.

## MEDICAL INTERVENTIONS AND PSYCHOSOCIAL CONSIDERATIONS

The transition process must address not only medical management but also the psychosocial issues specific to adolescents with CKD. Adolescence is a complex stage of life marked by physical, emotional and social changes that can be further complicated by the burden of chronic illness. CKD can directly or indirectly (through treatment) affect growth, puberty, self-esteem and social interactions, making tailored support during this period essential [3].

Adolescents often experience denial of their disease, which can lead to decreased adherence and follow-up. Ensuring a smooth and supportive transition is critical to maintaining patient engagement in nephrology care and minimizing the risk of loss to follow-up. This process requires addressing their evolving sense of identity, autonomy and responsibility for their health [3].

A key aspect of the transition is ensuring that patients understand the fundamental differences between paediatric and adult care. Paediatric care typically includes strong psychosocial support provided by parents, medical teams, psychologists and social workers, with legal guardians making most decisions. In contrast, adult care places full responsibility for medical decisions on the patient, requiring greater independence and self-management skills. In addition, access to non-pharmacological interventions, such as by dieticians, psychologists and physiotherapists, is often covered by public health systems in paediatric care but may require private insurance or out-of-pocket expenses in adult care, depending on the country. Patients need to be prepared for these changes as part of the transition process. This preparation can be supported through written materials, visual aids, interactive online tools or personalized counselling by trained professionals. Repeating key information during follow-up visits and applying teach-back methods can help confirm the patient’s understanding and readiness. Patient navigation tools or programs, when available, can also provide structured guidance and support for both patients and families throughout the transition process [5].

Effective multidisciplinary coordination is critical, especially for patients with genetic kidney diseases associated with extrarenal manifestations. Joint care protocols between paediatric and adult care teams need to be established in advance to ensure continuity of care. Kidney function monitoring is ideally complemented by the assessment and monitoring of extrarenal manifestations (depending on the disease), which requires input from multiple specialties, including endocrinologists, internists, general practitioners, orthopaedic surgeons, physical therapists, rheumatologists, neurosurgeons, otolaryngologists, ophthalmologists, audiologists, rehabilitation specialists, pain management specialists, haematologists, digestive specialists, urologists, geneticists or genetic counsellors or any other relevant specialists. This collaborative approach ensures holistic care tailored to each patient’s unique needs.

In addition to medical management, comprehensive sexual education is an essential component of the transition process. This includes information on contraception, prevention of sexually transmitted diseases and family planning in the context of a genetic disorder. Professionals such as psychologists, nutritionists, dentists, orthodontists, occupational therapists and social workers may also play a role in addressing the broader psychosocial and medical needs of these patients. Identifying risks such as depression or anxiety and providing early intervention are essential to supporting mental health during this critical period [2, 3, 6]. Because adolescents are often unaware of their legal rights or potential access to financial and social support, it is important to include this information explicitly during the transition process to support informed and autonomous decision-making. For an overview of key practical advice on medical and psychosocial aspects of transition, see Box 2.

Box 2: **Medical interventions and psychosocial considerations—practical advice.**

2.1. Patient education: Ensure the patient understands the disease, treatment options, the key differences between paediatric and adult care and the importance of a healthy lifestyle. This can be supported through written materials, visual aids, online tools, individual counselling or the use of patient navigation tools or a dedicated patient navigator to guide the patient and family through the transition process [2, 3, 5].

2.2. Psychosocial support: Address issues such as denial and non-adherence by providing psychosocial support, including early identification of mental health risks like depression and anxiety [3].

2.3. Sexual health education: Ensure that sexual health education is appropriately addressed by the care team, including topics such as contraception, prevention of sexually transmitted diseases and reproductive implications of genetic diseases [3]. While paediatric nephrologists may not always deliver this directly, they should ensure it is integrated into the broader care plan.

2.4. Multidisciplinary coordination: Involve specialists (e.g. ophthalmologists, ENT physicians, endocrinologists, geneticists, urologists) with pre-existing joint care protocols to ensure continuity of care for extrarenal manifestations [3].

2.5. Other support: Include paramedical professionals like nutritionists, occupational therapists, social workers and pharmacists as part of the care team. Additionally, facilitate engagement with patient associations for peer support and advocacy. Consider addressing patients’ awareness of their legal rights and available social or financial support, as this is often overlooked during transition and may impact their autonomy and access to care.

## GENETIC DIAGNOSIS AND ROLE OF THE GENETICIST IN TRANSITION

When transitioning patients with hereditary forms of CKD, whether confirmed or suspected, the medical geneticist or genetic counsellor is an essential part of the medical team, both during the paediatric and adult follow-up of the patient. During paediatric follow-up the role of the geneticist is both diagnostic and with possible direct implications for management and prognosis, genetic counselling being mainly addressed to the patient’s parents. In older children and adolescents, informing patients themselves is added to this role, with reinforming the patients playing a key role in a better understanding of the disease. In adult care, geneticists play a crucial role in familial planning, particularly for patients considering conception. This includes providing information about the inheritance pattern of the disease, the risk of transmission to offspring and potential reproductive options, such as preimplantation genetic testing or prenatal diagnostic testing, when applicable.

### Importance of genetic testing in transition

The rapid evolution of genetic testing technologies over the past few decades has significantly improved diagnostic capabilities. Next-generation sequencing (NGS) technologies, including copy number variation (CNV) analysis, enables parallel sequencing and analysis of multiple genes and variant types, increasing diagnostic yield. Functional studies have improved variant classification, refining pathogenicity assessment [7–9]. These advances underscore the importance of performing or re-evaluating genetic testing during the transition process for patients with suspected inherited CKD.

Inherited nephropathies often exhibit phenotypic heterogeneity, with certain features emerging gradually over time, this being one of the reasons why a clinical diagnosis can sometimes be challenging. A detailed phenotypic (re)evaluation and a comprehensive review of the family history are important components of the transition process. New information from these evaluations may refine or even change the presumed inheritance pattern or clinical diagnosis.

For patients who underwent genetic testing >3–5 years ago, it is important to assess the need for re-evaluation. Even in cases with an established genetic diagnosis, re-evaluation may add value, for example, to confirm that the genetic diagnosis fully explains the phenotype, to reassess treatment implications or to update recommendations for screening of family members considering the continuously evolving knowledge and development of new guidelines. Patients with highly suggestive clinical or familial traits but without molecular confirmation should always be (re)offered and prioritized for genetic testing, particularly in progressive CKD or conditions where early diagnosis can lead to targeted therapies, such as primary hyperoxaluria, cystinosis or Fabry disease. The re-evaluation may also be conducted by adult nephrologists when the patient enters their care, if the timing is more suitable then.

Although genetic testing is generally more relevant for nephrological management before the patient reaches kidney failure, this may lead to the misconception that its importance lies solely within the geneticist’s domain at later stages. However, in some hereditary kidney diseases, the genetic diagnosis can still influence nephrological care even after the initiation of dialysis or transplantation, particularly when there are extrarenal manifestations or when there is a risk of disease recurrence after kidney transplantation. Also, correct diagnosis is a patient right and comes with important implications for other family members. As the patient remains under nephrology care and may not have seen a geneticist for many years, it is the nephrologist’s responsibility to recognize the moment of transition as an opportunity to reinitiate genetic evaluation, not only for diagnostic purposes but also for patient education, family planning and assessing implications for other relatives. Continued collaboration between nephrologists and geneticists remains important throughout the patient’s care.

### Integration of genetic counselling and follow-up

Patients and families must be informed of the implications of genetic testing, including potential socio-economic implications (e.g. insurance eligibility). Negative results may warrant consideration of extensive genetic testing, such as whole exome sequencing (WES) or whole genome sequencing (WGS). Collaboration with or referral to a (nephro)geneticist can be considered to ensure adequate pretest and post-test counselling and interpretation. In addition, we advise that all unresolved cases of suspected hereditary nephropathies should be re-evaluated every 3–5 years given the continuing evolution of genetic testing technologies and reclassification of variants.

For patients who initially decline genetic testing, genetic counselling remains an important aspect of care even after the transfer. These discussions can be revisited in adulthood, especially before family planning, to ensure informed decisions.

The transition period can also be a moment to reflect on the potential impact of a genetic diagnosis for family members. When applicable, genetic testing can be considered for first-degree (adult) relatives if not already performed, as this can influence their own health monitoring with potential treatment consequences. Family members, especially minors, can consider monitoring treatable signs or symptoms, particularly if these have predictive value and may thus influence long-term courses, instead of performing a genetic test. Moreover, testing of family members can help assess eligibility for future living-related transplantation when applicable. Also, testing relatives is sometimes necessary to interpret the causality of genetic variants identified in the transitioning patient. Patients or their family members can be referred for genetic counselling to discuss the above.

### Ethical, cultural, legislative and religious considerations

Reproductive options, such as preimplantation genetic testing when available and applicable, ought to be presented transparently, along with their ethical dilemmas and regional, cultural and legislative variations. Religious beliefs may also influence patient decisions, particularly regarding contraception and fertility procedures. We recommend that these discussions involve the patient, the family when needed (based on the patient’s wishes and/or legal requirements for minors), the medical team and, if appropriate, a social worker or spiritual counsellor to ensure a holistic approach. As circumstances and perspectives may evolve, these discussions ought to be revisited later in adulthood.

### Visual overview of the genetic testing process

**Figure 1:**
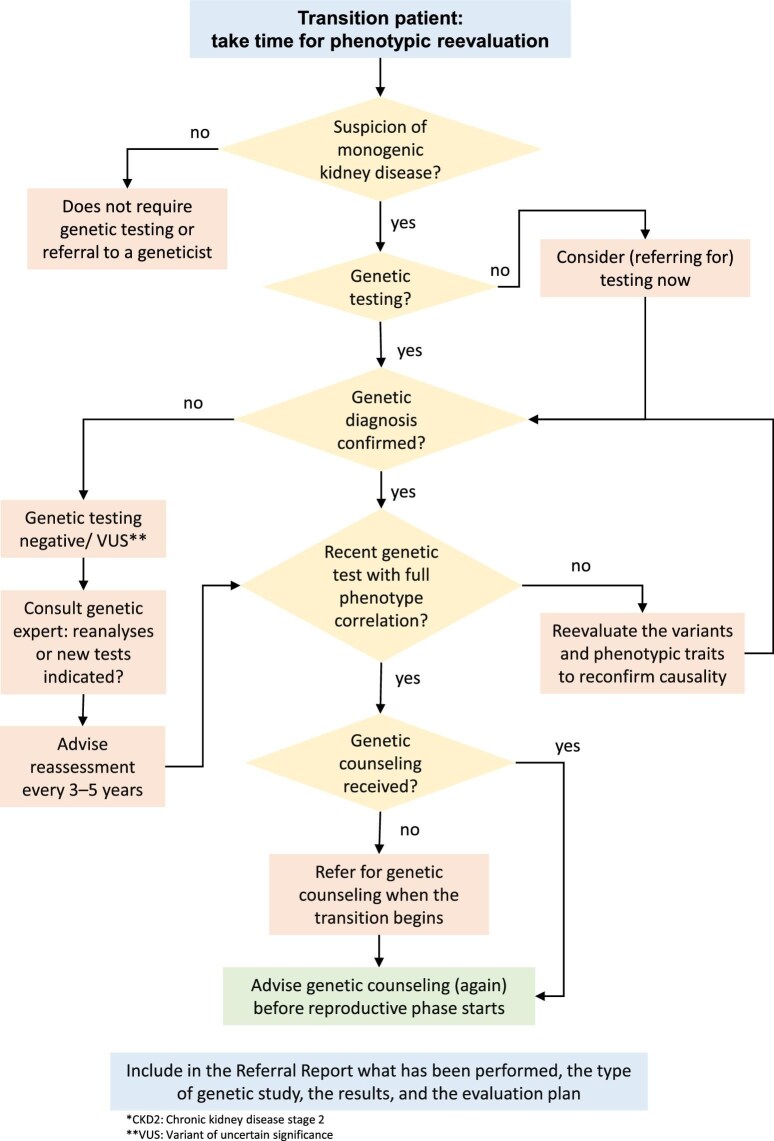
Schematic representation of decision-making processes involved in genetic diagnosis and counselling during the transition period. This flow chart provides a structured approach for evaluating the need for genetic testing, re-evaluating previous results and ensuring appropriate genetic counselling during the transition from paediatric to adult nephrology care. The diagram highlights critical steps such as identifying suspected monogenic kidney disease, assessing the validity and recency of genetic results, confirming phenotype–genotype correlation and integrating genetic counselling both at the beginning of transition and prior to reproductive planning. It emphasizes the importance of referring to a geneticist when test results are inconclusive or outdated and documenting all relevant findings in the referral report to the adult nephrologist.

To streamline the decision-making process during the transition, Fig. 1 provides a simplified flow chart that summarizes the key steps involved in assessing the need for genetic testing, re-evaluating prior results and ensuring timely genetic counselling. This visual representation ensures that no critical steps are overlooked.

For summarized practical advice on genetic evaluation and the role of the geneticist in transition, see Box 3.

Box 3: **Genetic diagnosis and role of the geneticist in transition—practical advice.**

3.1. Genetic counselling for family planning: Offer genetic counselling that includes reproductive options when appropriate, considering applicable laws, religious beliefs and shared decision-making. Ensure adequate time for education and informed decision-making. Emphasize addressing these issues prior to the reproductive phase [2, 3].

3.2. Ongoing geneticist involvement: Maintain geneticist involvement into adulthood, particularly when genetic findings have implications for long-term management, family screening or reproductive planning [3]. While the genetic cause may have less direct impact with age, it remains relevant for younger family members. The nephrologist is responsible for involving the geneticist when needed.

3.3. Ongoing genetic evaluation: Consider whether re-evaluation of the genetic diagnosis is warranted, especially if previous testing was performed >3–5 years ago. This may include reviewing whether the identified genotype still fully explains the current phenotype, taking into account new variant classifications or diagnostic technologies. Even in cases with a confirmed diagnosis, updated insights may have implications for prognosis, treatment or family screening. The time of transfer offers an opportunity for the adult nephrologist to reassess the clinical picture and, when appropriate, consult with a medical geneticist or consider further testing to support continuity of care and precision in management.

3.4. Phenotypic and family history assessment: As part of the transition process, perform a detailed phenotypic assessment and thorough family history review to refine or revise the clinical or genetic diagnosis, especially in patients with progressive CKD (KDIGO stage 2 or higher) or those with suspected hereditary nephropathies.

3.5. Management of inconclusive or negative results: For patients with negative or inconclusive genetic testing [e.g. variant of uncertain significance (VUS)], consult a (nephro)geneticist to determine whether advanced testing (e.g. WES, WGS) or reanalysis is needed. Re-evaluate negative results every 3–5 years to reflect advances in testing technologies and variant reclassification.

3.6. Follow-up for patients without genetic testing: Provide ongoing follow-up for patients who initially do not opt for genetic testing and revisit the discussion in adulthood, especially before family planning. Provide accessible genetic counselling tailored to the patient’s needs and circumstances.

## STRUCTURED TRANSITION PLANNING

A structured and individualized transition plan is essential for a successful transition from paediatric to adult nephrology care. To determine the patient’s readiness, it is crucial to confirm they understand their illness, evaluate their willingness to adopt new health-promoting habits and understand their future expectations regarding their condition and life plans.

However, we acknowledge that in many countries the timing of transfer to adult care is primarily determined by institutional or national policies and not always by the patient’s individual readiness. While early and well-prepared transitions are often considered beneficial, their implementation may be hampered by a lack of resources, personnel or coordination between services. In such contexts, the practical advice presented in this document should be flexibly adapted to local realities and applied as far as feasible to support the best possible outcome.

Tables 1 and 2 present sets of questions that can be posed to the patient and family to assess their level of acceptance and readiness for the transfer. These questions help to identify potential challenges and facilitators, allowing for a tailored transition process that meets the patient’s needs. Multiple consultations may be necessary to ensure that the patient can provide appropriate answers to these questions. If the patient has a complex condition that requires multiple input from multiple specialists or has an intellectual disability, social workers may need to be involved in the transition process [2].

**Table 1:** Suggested tool for evaluating patient readiness for transition and transfer to adult nephrology care.

Variables	Yes	Not yet
I am aware of the diagnosis of my illness		
I am aware of the long-term consequences of my illness		
I am aware of the physical limitations that my illness may cause		
I understand the significance of maintaining a healthy diet		
I am aware of the consequences of using drugs and alcohol		
I understand my treatment plan		
I am capable of administering my own treatment at the designated times		
I am aware of the potential negative effects of my treatment		
I am aware of my allergies		
I am willing to continue my follow-up in adult consultations		
I can independently obtain medications and schedule follow-up appointments		
Financial issues will not hinder my ability to continue my treatment and follow-up		
If I encounter any issues, I can seek assistance from my family or medical professionals		
I feel confident discussing my illness and treatment with a new doctor		

**Table 2:** Suggested tool for evaluating family/caregiver readiness for the patient’s transition and transfer to adult nephrology care.

Variables	Yes	Not yet
The patient is ready to take on the responsibility for the management of their medication and request follow-up consultations		
I am willing to relinquish responsibility for the patient’s health, medications and follow-up to the patient		
I am confident that the patient can manage their health and follow-up care independently		
I feel comfortable stepping back and allowing the patient to take control of their care		

The questionnaire will be reviewed by the paediatric nephrologist alongside the patient and family/caregivers to confirm the accuracy of the ‘yes’ responses. If any response is found to be inaccurate, the patient will be provided with tools and information to address them. In cases where the patient has a disability or special needs or special family circumstances, not all questions may be answered affirmatively. In such cases, the reasons for proceeding with the transition despite incomplete answers must be clearly documented in the transition report [2].

**Figure 2:**
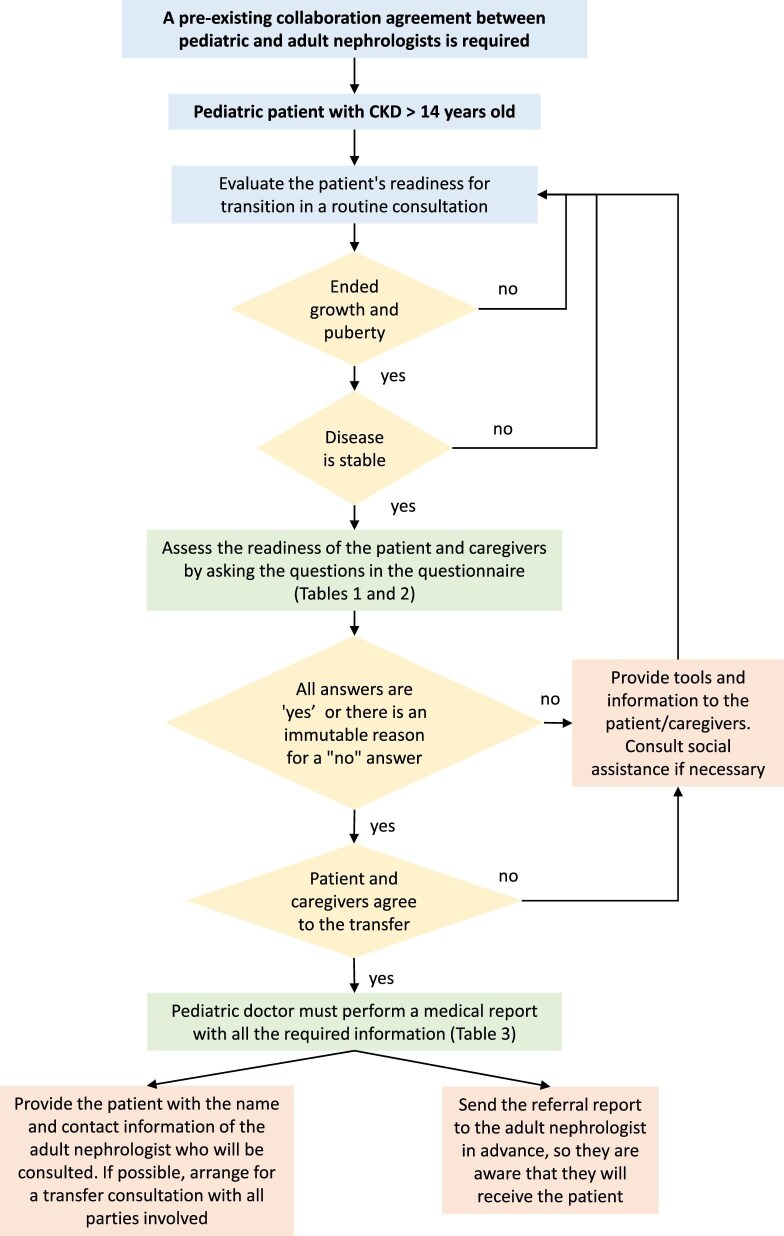
Algorithm for transitioning and transferring a patient from paediatric to adult nephrology care. This diagram outlines the structured process for evaluating and preparing paediatric patients with CKD for transfer to adult care. It includes readiness assessment through transition questionnaires (Tables 1 and 2), criteria for stability and growth completion and confirmation of agreement by both the patient and caregivers. The algorithm emphasizes the need for a detailed medical referral report (Table 3) and ideally an advance coordination with the adult nephrologist.

Fig. 2 provides a summarized flow chart of the structured transition planning process, outlining the steps from assessing the patient’s readiness for transition to coordinating with the adult nephrologist. This visual guide highlights critical elements such as use of the transition questionnaire (Tables 1 and 2), preparation of the medical referral report (Table 3) and post-transfer follow-up.

**Table 3:** Suggested content for the referral report to support the patient’s transfer from paediatric to adult nephrology care.

Patient identity information
Name Parents or caregivers Patient schooling level
Clinical data
Diagnosis Age at the time of diagnosis Personal background history Previous surgeries Include a copy of the latest laboratory and imaging tests Renal biopsy report (if performed)
Genetic data
Family history Genetic tests (include the date of testing, whether the result is considered a confirmed diagnosis, the genes analysed and relevant results including American College of Medical Genetics and Genomics classification: e.g. pathogenic variant, VUS) When there is a confirmed molecular diagnosis or a clear clinical diagnosis: the inheritance pattern Date when patient him/herself last received genetic counselling (and which centre)
Treatment history
Drug-related allergies Date of initiation and discontinuation of each medication, dosing, adverse effects, adherence, efficacy or lack of therapeutic efficacy
Assessments by other specialists
Which specialist are/were involved Summary of their findings and management
Long-term prognosis
Potential complications
Transition questionnaire and important considerations
The patient and caregivers have answered ‘yes’ to all questions in the questionnaire. If they have not, please provide a reason for the transfer at this moment. Does the patient or their caregivers have a disability? If yes, please explain. Does the patient or family have access to resources (e.g. transportation, financial aid) that may support a successful transfer? If not, please explain.
Contact information for the paediatric nephrologist in case of questions
Name and contact information (e.g. e-mail, phone number)

Once the actual transfer has been decided, a comprehensive medical report from the paediatric nephrologist is essential. This report includes the diagnosis, the performed genetic analyses and their results (if applicable), treatment history, progress and long-term prognosis. It also summarizes the responses to the transition questionnaire, noting any areas requiring stricter follow-up or unresolved questions, with clear explanations. Table 3 outlines the aspects to be included in the referral report to the adult nephrologist [4].

In addition, the transition report should ideally include the name and contact details of adult specialists familiar with the patient’s condition (e.g. nephrologists, geneticists or multidisciplinary teams with expertise in that specific disease). This is particularly relevant for patients with rare conditions requiring specialized follow-up or access to specific tests or treatments (e.g. cystine monitoring in cystinosis). Providing this information helps ensure continuity of disease-specific care, especially when the adult nephrologist taking over may not have direct access to such resources.

The first visit to a new adult nephrologist can be a stressful experience for the patient and family. Based on this, some hospitals have created a transition consultation where the patient, family members or caregivers and both paediatric and adult nephrologists are present. The objective is to introduce the patient to the new specialist responsible for their monitoring and to directly explain the medical history to the adult nephrologist. If this is not possible, it is recommended to provide the patient and family with the name and contact information of the adult specialist who will be handling their care.

Alternatively, when in-person joint visits are not feasible due to geographic distance, time constraints or limited resources, a virtual introduction (e.g. via video call) involving the patient, family and both the paediatric and adult nephrologists can be an effective option. This allows for an initial connection with the adult team and an opportunity to review key clinical information, helping ease the transition.

It is often during this first visit that the patient begins to build trust and a connection with the new physician. This visit also constitutes a significant adjustment for the patient’s parents or caregivers, who may experience uncertainty regarding their evolving role in the care process. For some adult nephrologists, management of an appointment that involves the patient, parents and themselves can be challenging, especially if they are unfamiliar with navigating these complex dynamics. It is recommended that this first visit include the patient’s parents to facilitate a smooth transition, while gradually introducing the concept that subsequent appointments will primarily involve only the patient and the adult nephrologist. This shift reflects the transfer from paediatric care, in which parents played a central role in decision-making and shared responsibility for the patient’s care, to adult care, where the patient assumes full autonomy.

To facilitate this important shift, another approach could be used during the late phase of the transition process, which would include having paediatric consultations with the patient seen alone, followed by joint discussions with the parents afterwards. This could ease the process of stimulating the patient’s autonomy and could potentially familiarize the patient with adult consultations before the first visits.

The first consultation ought to be longer than usual to allow the adult nephrologist to review the patient’s understanding of the disease, evaluate the effectiveness of ongoing treatments and answer any questions the patient may have. It is a common perception that the time invested into a longer first consultation will easily be regained during follow-up visits. A first consultation is also an ideal opportunity to re-evaluate the genetic aspects of the disease—especially if this has not been done recently by the paediatric nephrologist—and to perform a complete physical examination. If genetic testing has been performed, the medical record is expected to include the type of analysis performed, the genes analysed, the date of testing and the complete genetic report, if available.

In some cases, particularly when ultra-rare diseases are involved, adult nephrologists may not be as familiar with these specific conditions, especially those typically diagnosed and managed in paediatric settings, such as cystinosis or primary hyperoxaluria. Providing the adult nephrologist with advance notice of the patient’s consultation is essential, allowing them time to review relevant literature and update their knowledge on the specific condition.

To address this gap, it is important that adult nephrologists receive comprehensive training in the diagnosis and management of rare kidney and genetic diseases. This training includes advanced genetic testing, interpretation of genetic variants and the management of extrarenal manifestations, ensuring seamless continuity of care and tackling the unique challenges these conditions pose during the transition process.

An important component of this first consultation is the assessment of kidney function. Paediatric nephrologists use either the revised Bedside Schwartz formula or, more recently, the Chronic Kidney Disease in Children under 25 (CKiD U25) formula to estimate glomerular filtration rate (GFR), whereas adult nephrologists more commonly apply the Chronic Kidney Disease Epidemiology Collaboration equations. This change can lead to underestimation or misclassification of CKD severity in very young adults. In cases where estimated GFR values appear discordant or inconsistent with clinical findings, the use of age-appropriate formulas such as the CKiD U25 equation, Full Age Spectrum equation or combined creatinine–cystatin C equations may be considered to improve accuracy, particularly during the transition from paediatric to adult care [10–12]. In syndromes associated with low muscle mass, reliance on creatinine-based estimations alone may be misleading, and cystatin C–based or combined equations are preferable in these cases.

Parents remain an important source of support after the transfer and finding a balance between the roles of the adult nephrologist, the patient and the parents is essential for a successful transfer. A clear communication strategy and understanding of each party’s role can help to reduce the stress experienced by all and foster a collaborative environment that promotes the patient’s independence and well-being.

Details of the transition questionnaires and referral report are provided in Tables 1–3. For practical guidance on structured transition planning, see Box 4.

Box 4: **Structured transition planning—practical advice.**

4.1. Transition questionnaire: Consider using a detailed questionnaire to assess patient and family readiness for transition, including their understanding of the disease, willingness to adopt new health-promoting habits and expectations regarding their condition. Address any areas of concern with appropriate interventions and document unresolved issues in the transition report. The proposed questionnaires (Tables 1 and 2) are examples and can be adapted to individual cases, cultural contexts and country-specific health systems. In addition, many other validated questionnaires are available in different languages and can also be used [2].

4.2. Referral report: Provide the adult nephrologist with a comprehensive referral report. It is recommended to include the diagnosis, treatment history, progress, long-term prognosis, details of any genetic analysis performed (e.g. type, genes analysed, date and genetic report) and any unresolved questions or concerns from the transition questionnaire [4].

4.3. First consultation structure: Ensure that the initial visit with the adult nephrologist is longer and includes a complete review of the patient’s history, physical examination and discussion of treatment. In the case of inherited CKD, a review of genetic data is recommended. This visit is ideally conducted by an experienced consultant nephrologist. Kidney function assessment is advised using age-appropriate formulas to reduce the risk of misclassification [10–12].

4.4. Genetic aspects during transition: Review the practical advice concerning genetics, covering genetic counselling, genetic testing and its implications for long-term care and family planning.

4.5. Coordination between the paediatric and adult nephrologists: Ensure effective coordination with a designated adult nephrologist prior to transfer. Allow sufficient time for the adult nephrologist to review the patient’s condition, especially for rare conditions that may require additional preparation.

4.6. Transfer consultation: If possible, schedule a transfer consultation with the paediatric nephrologist, adult nephrologist, patient and family. This meeting will introduce the patient to the new specialist and ensure a smooth transition of care as well as transfer of information. If a meeting is not possible, provide the patient and family with the name and contact information of the adult nephrologist. Either way, the adult nephrologist should receive a transfer letter that includes the entire medical history of the patient.

4.7. Training for paediatric and adult nephrologists: It is important that both paediatric and adult nephrologists receive appropriate training in the diagnosis and management of rare kidney and genetic disorders. This includes familiarity with advanced genetic testing, interpretation of genetic variants and management of the extrarenal manifestations often associated with these conditions.

4.8. Role of parents in the transition/transfer process: Parents have an important role to play during and after the transfer, providing emotional support and continuity. As the focus shifts to patient autonomy, involving parents in discussions to define their evolving role helps ensure a gradual and balanced transition that supports both the patient and the care process.

## SPECIAL CONSIDERATIONS FOR SPECIFIC DISEASES

Certain inherited or inborn nephropathies require a tailored approach to the transition process due to their unique clinical, genetic and management challenges. Below, we outline specific considerations for selected inherited kidney diseases, highlighting the need for individualized transition plans.

### Alport syndrome

Alport syndrome is associated with a wide phenotypic spectrum depending on the gene involved (*COL4A3, COL4A4, COL4A5*) and the type of genetic variant. The transition report is expected to include detailed genetic test results that specify the affected gene(s), the type of variant(s) and the inheritance pattern. This information is essential to stratify prognosis and tailor follow-up strategies in adult care.

Males with X-linked Alport syndrome, as well as individuals with autosomal recessive or digenic forms, have the highest risk of progressive CKD. In contrast, autosomal dominant forms (previously misnamed thin basement membrane disease and now considered part of the Alport spectrum) often have a milder course but still warrant careful follow-up, as 10–15% of these patients are considered at risk for progressive disease [13].

Genetic testing can be considered for first-degree (adult) relatives, if not already performed, as this can influence their own health monitoring with potential treatment options. Furthermore, it can help assess eligibility for future living-related transplantation. As discussed earlier, these considerations are relevant in many hereditary kidney diseases but are particularly important in Alport syndrome due to the implications for both clinical management and donor selection. Importantly, current recommendations, including those from the European Rare Kidney Diseases Reference Network (ERKNet) 2024 guidelines, discourage in most cases the use of heterozygous *COL4A3* or *COL4A4* carriers as kidney donors [13, 14].

The transition report is recommended to include the age of onset of microalbuminuria and whether renin–angiotensin–aldosterone system (RAAS) blockade with angiotensin-converting enzyme inhibitors (ACEIs) has been initiated. Guidelines support initiating ACEIs in all males with X-linked Alport syndrome and in both males and females with autosomal recessive forms after the age of 2 years when microscopic haematuria is diagnosed, regardless of proteinuria [14]. In autosomal dominant forms and in females with X-linked Alport syndrome, ACEI should be started after microalbuminuria is detected, although recent studies recommend ACEI when specific genetic variants are involved, even before the onset of overt microalbuminuria [15]. New molecules with exciting results on adult cohorts of patients, such as sodium–glucose co-transporter 2 inhibitors (SGLT2is), could be considered in addition to RAAS inhibitors in patients with albuminuria. This timing is relevant for adult follow-up and should be clearly documented in the referral report, as recommended by the ERKNet 2024 guidelines [14]. It is important to begin discussing the teratogenic potential of ACEIs and angiotensin receptor blockers (as well as other drugs) during puberty in the presence of the parent(s) and to continue reinforcing this information at subsequent visits.

As recommended by the ERKNet 2024 guidelines, audiological and ophthalmological evaluations should begin in childhood and continue into adulthood [14]. Therefore, it is advisable to include recent audiological and ophthalmological evaluations in the referral report, as these extrarenal manifestations often require coordinated follow-up in adult care.

### Autosomal dominant polycystic kidney disease (ADPKD)

ADPKD, the most common inherited cystic kidney disease, requires specific attention during the transition process due to its progressive nature and potential for extrarenal complications. Details of genetic testing are important to include in the referral report, as they provide prognostic information and can guide the frequency and intensity of follow-up in adult care.

For patients without genetic testing but with a clinical diagnosis based on family history and imaging findings, it is advisable to consider re-evaluating the possibility of genetic testing during transition. Although a genetic diagnosis is not required to confirm ADPKD, it may offer significant benefits, including genetic counselling, clarification of inheritance patterns and evaluation of potential related donors, particularly in the context of living kidney transplantation.

It is important to clearly document any positive family history of cerebral aneurysms or sudden death, as this information may indicate the need for magnetic resonance angiography screening [16].

From the age of 15 years onwards, magnetic resonance imaging–based renal volumetry may be performed to estimate total kidney volume, which allows for classification using the Mayo ADPKD imaging-based risk stratification tool. This classification is useful both for follow-up and for informing therapeutic decisions. Although tolvaptan is currently approved for use in individuals ≥18 years of age, early risk stratification in adolescence can help identify candidates who may benefit from closer monitoring and facilitate timely initiation of treatment once they become eligible [17, 18].

Abdominal imaging should include the liver. Liver cystogenesis generally occurs later than kidney cyst development, and early detection of multiple liver cysts in young women should prompt against the use of oestrogen-based oral contraceptive therapies. While recent Kidney Disease: Improving Global Outcomes (KDIGO) guidelines no longer broadly restrict their use, they advise caution in patients with moderate–severe polycystic liver disease (PLD), in whom oestrogen-containing therapies should be avoided. In contrast, such therapies can be used under supervision in individuals with mild or no PLD [16].

### *HNF1B* nephropathy

*HNF1B*-associated nephropathy is characterized by significant phenotypic variability and incomplete penetrance, making clinical management and family counselling particularly complex. A detailed family history is valuable, focusing on kidney anomalies, diabetes mellitus and impaired glucose metabolism, which are common manifestations of the disease [19].

It is advisable to regularly monitor serum glucose, haemoglobin A1c and pancreatic enzymes and to involve endocrinology specialists when anomalies are detected or suspected. Pancreatic anomalies, including endocrine or exocrine insufficiency, may be present and complicate the clinical course [19, 20].

Genital malformations are also frequently observed, and pelvic ultrasound in females, along with fertility assessment in both sexes, when relevant, can aid in early identification and supportive care.

Genetic counselling is particularly important to explain the high phenotypic variability and incomplete penetrance within families, as these factors make it difficult to predict the severity of potential disease in future children.

In transplant patients, regular monitoring for diabetes mellitus is essential given the increased risk associated with immunosuppressive regimens based on steroids and calcineurin inhibitors such as tacrolimus [21].

### Congenital anomalies of the kidney and urinary tract (CAKUT)

CAKUT represents a diverse group of structural anomalies that often require coordination between nephrologists and urologists during the transition process. Given their greater expertise in these conditions, paediatric urologists are encouraged to collaborate with their adult counterparts to ensure a smooth transition of care.

Genetic evaluation may be considered not only for syndromic cases but also for any patient with CAKUT and CKD identified during childhood. A referral to a medical geneticist may assist in determining the most appropriate testing strategy, particularly in cases of unclear aetiology or suspected syndromic associations. CAKUT serves as an example of a phenotype group where genetic testing strategies continue to evolve, and the time of transition can be an opportunity to reflect on whether testing is appropriate in each individual case.

### Nephrotic syndrome

Nephrotic syndrome encompasses a spectrum of disorders with different genetic and clinical characteristics, including steroid-sensitive nephrotic syndrome (SSNS), steroid-resistant nephrotic syndrome (SRNS) and congenital nephrotic syndrome (CNS). Each subtype has unique challenges in the transition from paediatric to adult care. Each subtype of nephrotic syndrome requires tailored transition protocols to optimize care, including updating genetic testing, monitoring for relapses and adapting treatment plans to adult care settings.

Continued monitoring for steroid-related complications, such as obesity, hypertension and growth impairment, remains crucial during and after transition [22]. Screening for anti-nephrin antibodies, where available, may help distinguish non-genetic forms of nephrotic syndrome. Recent evidence suggests these antibodies are present in a significant proportion of patients with SSNS, providing a useful tool to support disease classification and guide immunosuppressive treatment decisions [23].

SRNS demands close collaboration with medical geneticists, as monogenic forms are common and have important implications for treatment decisions, risk of recurrence after transplant and family counselling. Adult care should focus on monitoring for drug toxicity from calcineurin inhibitors or other immunosuppressive agents used in paediatric management. Discussion of advanced therapies, such as monoclonal antibodies, may be necessary during and after the transition period [24].

At the moment of transition, involvement of specialist centres remains crucial for patients with CNS, who are typically on kidney replacement therapy by this stage. These centres can ensure continuity of care, reassessment of genetic findings and multidisciplinary coordination to manage long-term systemic complications and support transplant outcomes.

### Primary hyperoxaluria

Primary hyperoxaluria (PH) is a rare autosomal recessive disorder characterized by excessive oxalate production, leading to kidney stones, nephrocalcinosis and kidney failure in >50% of patients. PH patients with CKD stage G4–G5 may develop life-threatening systemic disease due to systemic oxalate deposition affecting organs such as the bones, heart, vessels, nerves and eyes.

During the transition from paediatric to adult nephrology care, it is essential to assess whether the patient is a candidate for RNA interference therapies such as lumasiran or nedosiran, particularly in PH type 1 (PH1), where these treatments have shown significant efficacy in reducing oxalate levels [25]. However, important unresolved questions remain, such as the safety and implications of small interfering RNA therapy during pregnancy. The transition and transfer period will be the moment when these questions will probably be addressed.

It is also important to assess B6 responsiveness, as this cannot be reliably predicted by genotype alone. Genetic testing remains essential for accurate diagnosis and subtyping, but biochemical monitoring is required to evaluate treatment response [25]. For PH1 patients on RNA interference therapy or pyridoxine, monitoring of urinary oxalate and glycolate is necessary in those with preserved kidney function, while plasma oxalate is more informative in patients with advanced CKD. Both measurements carry important limitations: urinary oxalate excretion may vary by >40% within the same patient from day to day and interpretation of plasma oxalate requires knowledge of the analytical method used and the patient’s kidney function stage [26, 27].

Special considerations during transition include ensuring that the adult nephrologist is familiar with PH-specific management strategies, such as intensive hydration, dietary counselling and the specific transplantation strategy. Coordination with multidisciplinary teams, including geneticists, urologists and dietitians, as well as experts from a reference centre, is essential to address the unique challenges posed by this condition.

Management can be especially challenging in patients with kidney failure. Conversely, patients with seemingly mild disease may experience a sudden decline if not managed appropriately. A key decision in PH1 patients with end-stage kidney disease is whether to pursue kidney transplantation alone (in cases of proven efficacy of RNA interference therapy or pyridoxine) or combined liver–kidney transplantation, either directly or sequentially performed. This choice must be personalized, taking into account the full clinical context and ideally involving consultation with experts in PH.

Embedding patient care within expert networks and ensuring regular re-evaluation of disease status and treatment response are strongly advised to optimize outcomes and anticipate complications across the transition period.

### Cystinosis

Cystinosis is a rare autosomal recessive lysosomal storage disorder characterized by the accumulation of cystine in various organs and tissues, leading to progressive renal and extrarenal manifestations. Early symptoms, such as Fanconi syndrome, typically appear within the first year of life, while extrarenal complications, including ocular problems, hypothyroidism, myopathy and neurocognitive problems, develop during adolescence and adulthood.

The transition of cystinosis patients from paediatric to adult care requires a multidisciplinary approach involving nephrologists, metabolic specialists and other subspecialists. Key considerations during this transition include managing adherence, monitoring systemic complications and managing fertility issues, as cysteamine treatment must be discontinued during pregnancy. Coordination between paediatric and adult care teams is essential to ensure continuity of care, particularly in managing the systemic and progressive nature of the disease [28].

It is important to establish an individualized transition plan that includes psychological support, education about treatment regimens and regular monitoring to optimize long-term outcomes.

An additional complexity arises when patients undergo kidney transplantation, as the underlying systemic cystinosis continues to progress, requiring dual follow-up—one nephrologist managing the kidney transplant and another in a multidisciplinary team overseeing cystinosis-related complications and monitoring cysteamine treatment that should be continued life-long. Alternatively, a dedicated nephrologist with expertise in both transplantation and this ultra-rare disease may be needed for comprehensive care.

## SUMMARY AND CONCLUDING REMARKS

For this article the working groups for genetic kidney diseases of the two large scientific European societies in nephrology, the European Renal Association and the European Society for Paediatric Nephrology, joined forces to present practical advice on the transition for patients with inherited kidney diseases. Given the improved therapeutic possibilities in neonatology, paediatric intensive care and paediatric nephrology, prognosis for patients suffering from these diseases has been improving, with more patients reaching adulthood. Still, many of these patients have an extensive personal medical history requiring special attention. To guide implementation of transition strategies, we outlined key practical advice. However, both knowledge gaps and important infrastructural challenges remain and will require further attention in the future. Reimbursement of the elements of transition is unfortunately not funded in many national health systems. Therefore, all efforts should be made to implement the described aspects in routine reimbursement. As genetic medicine is increasingly recognized as an ongoing component of care—relevant not only for diagnosis but also for information on prognosis and treatment, reproductive options and testing or screening of relatives—its integration into transitional care is essential and clear strategies are required for these lifelong conditions.

## Data Availability

All relevant references and materials used in the development of this practical advice document are cited in the text.
